# Steering towards success in stormy times: *FEBS Open Bio* in 2021

**DOI:** 10.1002/2211-5463.13058

**Published:** 2021-01-04

**Authors:** Miguel A. De la Rosa

**Affiliations:** ^1^ FEBS Open Bio

## Abstract

In this Editorial, the Editor‐in‐Chief Professor Miguel A. De la Rosa discusses the performance and development of *FEBS Open Bio* in 2020 and outlines his plans for the journal in 2021.

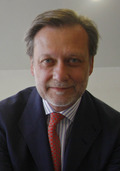

Any summary of 2020 will necessarily focus on the devastating effects of the coronavirus pandemic and the effects of lockdowns worldwide. The pandemic and the response have created a whole host of challenges, which have affected the research landscape in a myriad of ways – researchers have found themselves unable to enter their laboratories, preventing them from completing critical experiments. Students have found their graduation plans postponed, careers have been put on hold, and many people have had to juggle work commitments and childcare as schools and nurseries closed. More widely, healthcare workers and health services have been put under enormous strain, and the effects of lockdowns on industry, the economy, and physical and mental health are likely to be considerable.

As an online‐only journal, *FEBS Open Bio* has been able to continue operations, but has still experienced substantial challenges. Our Editorial Office in Cambridge was closed, and our staff have had to adapt to working from home. Many members of our Editorial Board had to transition to online teaching at short notice, which took up all available time. The clinicians on the board had to focus on treating patients, making them unavailable to handle manuscripts. Likewise, it became increasingly difficult to find willing reviewers for our submissions as researchers had to prioritise their own work. The 45th FEBS Congress, to be held in Ljubljana, was postponed to 2021, delaying plans for cross‐promotion between *FEBS Open Bio* and the Congress. Furthermore, the journal's first ever Editorial Board meeting had to become an online‐only event.

The challenges above were complicated further by the phenomenal growth the journal experienced in 2020 – by the end of November, our submissions had increased by an amazing 72% as compared to the same period in 2019. In addition, our accepted manuscripts increased by 60% in the same period. In the face of such a large increase in submissions and acceptances, our Editorial Office staff and Editorial Board worked tirelessly to ensure that we continued to process manuscripts and address author queries in a timely manner. I discuss the measures taken to facilitate the discovery of willing reviewers and reduce the time taken to decision below. While the increase in submissions may partially be driven by laboratory closures leaving researchers little to do but write up completed work, submissions to *FEBS Open Bio* had already been increasing year on year long before the pandemic began and may be a result of increased awareness of the journal, our prestigious Editorial Board and our reputation for publishing sound science across biochemistry and the molecular life sciences.

## Welcome to the new members of the Editorial and Editorial Advisory Board

In October 2020, we welcomed Vessela N. Kristensen as the 39th member of our Editorial Board.

**Vessela Kristensen**

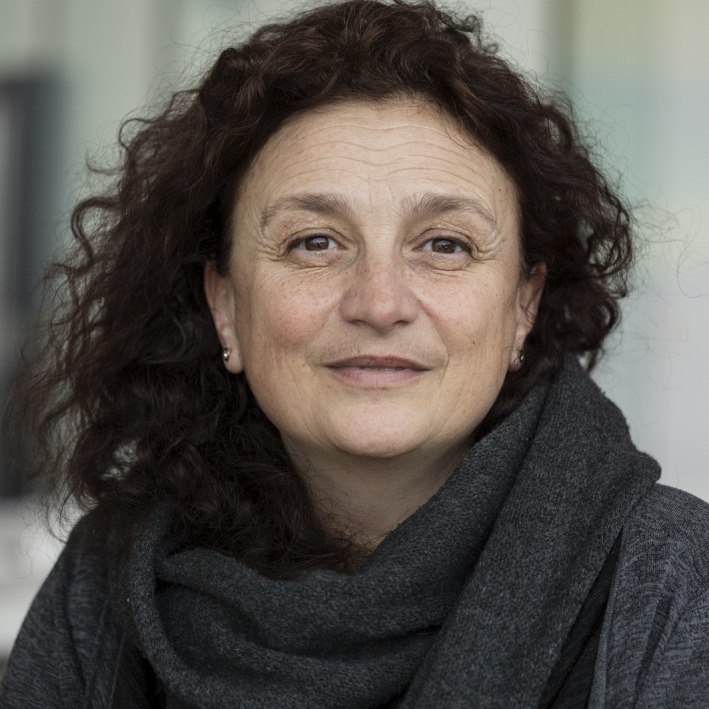

The scientific career of Vessela N. Kristensen started in the field of toxicology. Her PhD thesis encompassed the functional characterisation of polymorphic CYP at the Karolinska Institutet to elucidate the effect of genetic polymorphisms and their relevance to environmentally induced cancers. During her postdoctoral work, Kristensen studied aromatase (CYP19) in the laboratory of Dr. Nobuhiro Harada in Japan. Later technology allowed her to expand her studies of how genetic variation affects the occurrence of somatic alterations, gene expression patterns and genome‐wide copy number alterations in human breast at a whole‐genome scale. As a professor at the Faculty of Medicine, her projects have become translational in nature. Kristensen has been guest professor at the NCI, NIH in 2003 and Princeton University in 2012. As Director of Research at the Department of Medical Genetics at Oslo University Hospital, Kristensen's research interests have expanded to the genetic basis of other diseases and have turned to the functional effects of disease‐causing mutations. Vessela Kristensen leads large international networks in the framework of EU ERA CoSysMedicine and Horizon 2020. For this overall contribution to cancer research, she has received the Mørk legat prize in 2012 and the King Olav's award for cancer research in 2018. In 2020, she became a member of the Norwegian Academy of Science.

Vessela Kristensen joins Cornelia de Moor, Irene Díaz‐Moreno, María Fabiana Drincovich, Alexander Gabibov, Sergio Grinstein, Gabor Juhasz, Alicia Kowaltowski, In Hye Lee, Marcelo López‐Lastra, Ivana Novak, Rafael Radi, Josep Rizo, Lena Ruiz‐Azuara and Guangbiao Zhou as the 15th member of the Editorial Board appointed in 2020. Biosketches of all of them were published in a couple of former editorials in 2020.

Moreover, Beáta G. Vértessy, who has served on the Board since 2013, has been promoted to Senior Editor. I would like to offer a warm welcome to Vessela and congratulate Beáta on her promotion. I am especially thankful to all our formerly appointed senior and associate editors, many of whom have served on the board since the journal launched in 2011.

In addition, 2020 saw the introduction of our Editorial Advisory Board, which at present has 27 members. The Editorial Advisory Board are committed to providing peer review for manuscripts in their field of expertise, thereby ensuring that the journal is well‐positioned to review submissions in a thorough and timely manner. The 12 inaugural members were swiftly joined by Janesh Kumar [National Centre for Cell Science (NCCS), Pune, India] and Chongde Sun [School of Agriculture and Biotechnology, Zhejiang University, China] in April, and since then, we have appointed another 13 members. I am pleased to announce the appointment of the following members of our Editorial Advisory Board and welcome them to the journal:

- **Alexandre E. Benedetto**
  Division of Biomedical and Life Sciences, Lancaster University Faculty of Health and Medicine, United Kingdom
- **Usa Boonyuen**
  Faculty of Tropical Medicine, Mahidol University, Thailand
- **Vicki Clifton**
  Mater Research Institute, The University of Queensland, Brisbane, Australia
- **Fabiana Geraci**
  Department of Biological, Chemical and Pharmaceutical Sciences and Technologies (STEBICEF), University of Palermo, Palermo, Italy
- **Ramon Grima**
  University of Edinburgh, UK
- **Bernadett Kalmar**
  University College London (UCL), Queen Square Institute of Neurology, London, UK
- **G.W. Gant Luxton**
  University of California‐Davis, USA
- **Jonathan Martinez‐Fabregas**
  Cell Signalling and Immunology, School of Life Sciences, University of Dundee, Dundee, UK
- **Heinz Peter Nasheuer**
  School of Natural Sciences Centre for Chromosome Biology, NUI Galway, Galway, Ireland
- **Sandra Romero‐Córdoba**
  National Institute of Medical Science and Nutrition SZ (INCMNSZ), Mexico City, Mexico
- **Constantine A. Simintiras**
  Interdisciplinary Reproduction and Health Group, University of Missouri, Columbia, USA
- **Suman Thakur**
  Centre for Cellular and Molecular Biology, Hyderabad, India
- **Yueh‐Hsun Kevin Yang**
  The City University of New York ‐ The City College, USA

## The journal continues to grow

As noted in the Introduction, submissions and acceptances increased dramatically in 2020 as compared to 2019. This is fantastic news, and I am pleased that so many authors are considering our journal for the publication of their work. We have published several excellent papers this year, including a characterisation of a novel cold‐adapted transglutaminase from Atlantic cod, which may have implications for food processing [1]. This article was the subject of our first commentary article [2], and we hope to commission commentaries to accompany other papers of especial significance in the future. Other exciting work across the molecular and cellular life sciences published in *FEBS Open Bio* this year includes a report of alterations in neuronal development in a mouse model of Timothy syndrome [3], the crystal structure of botulinum neurotoxin subtype A3 cell‐binding domain in complex with the ganglioside GD1a [4], engineered variants of tobacco etch virus protease with enhanced enzymatic activity [5], the discovery that autologous apoptotic neutrophils inhibit inflammatory secretion by dendritic cells [6] and the use of fluorescence microscopy to reveal cooperative binding of cardiac troponin and tropomyosin to filamentous actin [7]. These are but a few of the fascinating studies published this year in the journal.

The research topic of single greatest interest this year is without question the SARS‐CoV‐2 pandemic. It is therefore perhaps unsurprising that our most downloaded and most cited article published in 2020 reports on a structure‐based virtual screen that identified 28 bioactive compounds that may have potential for the development into anti‐SARS‐CoV‐2 targets [8]. A second study, published later in the year, reported that the expression of five genes known to encode coronavirus receptors is differentially expressed between cancer and control tissues and is associated with COVID‐19 comorbidities [9]. These two *in silico* studies demonstrate the value of large datasets for rapid identification of potential drugs and gene targets for disease treatment.

The journal's Education section, introduced in 2017 under the helm of Angel Herráez and Luciane V. Mello, also continues to go from strength to strength. In 2020, we published five Education articles, on subjects as diverse as how to provide good written feedback to students [10], a study of how teaching laboratory peer groups affect student attainment [11] and recommendations for teaching biologically inspired design [12]. In addition, a total of 10 Education articles were submitted to the journal, our most in a single year to date. Many of our education articles are well‐downloaded, indicative of their interest to educators. Like all of our articles, the papers in the Education section are free to read. Moreover, our Education articles are published at no cost to the authors, with FEBS supporting the costs as part of our commitment to supporting biochemistry education.

*FEBS Open Bio* is a signatory of the San Francisco Declaration on Research Assessment (DORA) and uses a range of metrics to evaluate both its own performance and the individual impact of the articles it publishes. Journal performance improved in 2020 based on several metrics. Our median times to first and final decision have decreased in 2020 as compared to 2019 (as discussed further below), in line with our commitment to providing rapid and thorough peer review. Furthermore, downloads of our articles continue to rise dramatically: in addition to the articles mentioned above, other well‐downloaded articles this year include a report that the histone demethylase KDM3B protects against ferroptosis via SLC7A11 [13] and a study of the role of HSF1 in the mitochondrial unfolded protein response [14]. The authors are free to post the final version of record of their article in any repository they like, thereby helping to boost the visibility of articles published in *FEBS Open Bio*. This is an important benefit of the open‐access publishing model for authors. Several of our articles received considerable attention online, which is quantified as an Altmetric Attention Score. Unsurprisingly, our two articles on SARS‐CoV‐2 saw the most engagement online [8, 9]. Our Education articles also received high online attention scores [10, 11, 12], as did studies on the effect of endurance exercise duration on muscle hypertrophy [15] and potential sources of interference with the detection of alpha‐synuclein seeds by the qRT‐QuIC technique [16]. Several journal‐level bibliometrics for 2019 increased from 2018, including the two‐ and five‐year impact factor (both 2.231 for 2019) and CiteScore (3.000 for 2019). Our highest cited article from the last two years reports on the use of the MinION™ nanopore sequencer to identify bacteria based on 16S rRNA genes [17].

## The journal's first Editorial Board meeting (was held online)

To mark the beginning of my tenure as Editor‐in‐Chief, it was agreed in 2019 that we would host the first Editorial Board meeting for *FEBS Open Bio* in Seville in late 2020. Preparations began in early 2020 as we explored possible venues and consulted with our editors to determine the optimal date. As the coronavirus spread and travel restrictions began, it became apparent that we needed a backup plan in the unlikely (as it seemed at that time) case it would be impossible to hold a physical meeting in the autumn. As the months passed, it became increasingly clear that 2020 was not going to be a year for travel, and the decision was made to make the meeting a virtual event.

This may have been a blessing in disguise, however, because many editors who were unable to attend the physical meeting were able to log on to the virtual event, making for a very fruitful and active discussion. The meeting covered a range of topics and featured talks by the Chair of the FEBS Publications Committee László Fésüs, the Image Integrity Analyst Jana Christopher, the journal's Editorial Manager Duncan Wright and me. The meeting was also attended by Mary Purton, the FEBS Press Publisher, and Jackie Jones, publisher at Wiley, our publishing partner. Jackie Jones answered our editors' questions on developments in open access and the implications for authors in low‐income countries. The journal's history and recent performance were described by Duncan Wright and me, and an overview of FEBS and its publishing history was presented by László Fésüs.

Jana Christopher introduced the members of our Editorial Board to her important role as Image Integrity Analyst during the meeting. *FEBS Open Bio* and the other three FEBS Press journals take image integrity and the issue of paper mills very seriously (as discussed in recent articles by Jana Christopher [18, 19]), and screen all accepted manuscripts for potential figure issues. This service, which is performed at no extra cost to the authors, helps identify both genuine mistakes (such as the accidental duplication of a panel) and anomalies with potentially more sinister origins. Such detection can save the author (and the journal) an embarrassing corrigendum in the event of a mistake, as well as helping ensure that manuscripts with fraudulent data are not published, preventing them from contributing to the reproducibility crisis. The time, work and expertise required means that few other journals routinely perform figure checking, and I consider this service helps distinguish *FEBS Open Bio* above its competitors.

Several key conclusions were reached during the meeting. We have decided that, henceforth, the authors will be required to recommend at least three and up to five reviewers at the time of submission. Formerly, this was optional, but in the face of increasing difficulties in finding reviewers, we have decided to make it compulsory. There was spirited discussion on the journal's minimum standards for peer review; while we will remain true to *FEBS Open Bio*'s core mission of considering sound science, we now expect that all submissions will represent a minimum contribution to the relevant field, so as to filter out offerings from paper mills (which take advantage of mega journals by preparing (potentially fraudulent) manuscripts based on minor derivations of published work) and cases of extreme ‘salami slicing’, where the work is very simple/preliminary. We hope that this decision will allow us to continue to serve the community by providing a home for sound science while protecting the reputation of the journal and the integrity of the scientific record [19].

Several editors voiced the hope that we could hold annual online Editorial Board meetings, such was the appreciation of the merits of the format. I consider the meeting a great success, and now that we are used to the technology, anticipate that we will be able to hold even smoother and more interactive meetings online in future. I am grateful to our presenters, our Editorial Board and Mary Purton for hosting the meeting, Duncan Wright for arranging it and preparing the minutes, and Jacob Weller for excellent technical support and for monitoring the chat.

## Reducing the time to decision

With the increase in global research output, many journals are experiencing increasing difficulties in finding willing reviewers. For a growing mega journal like *FEBS Open Bio*, which considers diverse submissions across the molecular and cellular life sciences, this problem is particularly acute. Moreover, the pressures on researchers caused by the coronavirus pandemic have exacerbated this problem even further.

To ensure that we can better fulfil our goal of facilitating rapid peer review of submissions, we have introduced a number of new initiatives. The journal has a large pool of volunteer reviewers, which we have sought to strengthen and consolidate by contacting all reviewers to ensure they remain active and committed to reviewing manuscripts for the journal, and by ensuring that all volunteers have up‐to‐date research keywords in our peer‐review system. Moreover, 2020 saw the introduction of the journal's Editorial Advisory Board, which has 27 members on hand to assist editors with reviewing manuscripts where needed. Finally, to reduce the burden on overwhelmed editors and also reduce the time to decision for authors, we are now swift to reassign manuscripts if editors are unable to act within 10 days.

These initiatives appear to be paying dividends, with median time to first decision dropping from 37 days in 2019 to 33 days in 2020, median time to final decision from 69 to 63 days and median time to acceptance from 98 to 94 days. While reducing time to decision is important in an age of rapid research advances, it remains a secondary to *thorough* peer review – ultimately, we work hard to ensure that authors receive careful, comprehensive and constructive comments with which to improve their work for publication. Quality will not be sacrificed for speed.

## Future developments

Last year saw a number of new developments for the journal, but we still have several new plans in the pipeline. I am pleased to announce the creation of a new section for *FEBS Open Bio* called ‘In the Limelight’. This section will feature a small number of review articles on an exciting research area. In 2021, we will publish two such sections, one on bioplastics with Guest Editor Raffaele Porta. This section will accompany the ‘Plastics: revolution, pollution and substitution’ special section to be held at the 2021 FEBS Congress (Ljubljana, Slovenia). The second ‘In the Limelight’ section will focus on membrane‐less organelles with Guest Editor Irene Díaz‐Moreno, to accompany the International Symposium on Membrane‐Less Organelles in Cell Life and Disease (Seville, Spain), which has been rescheduled to this year. Look out for these sections in an upcoming issue.

We intend to promote the journal and its prestigious Editorial Board through a series of interview articles with our editors. This series is being managed by our new Senior Editor Beáta G. Vértessy with support from Editorial Manager Duncan Wright. Beáta will be the subject of the first article, which will be published in a future issue. In addition, we hope to promote articles of especial interest through one of two new article types: commentaries, to be commissioned for articles that demonstrate a significant advance in the field, and lay summaries for articles with implications for the general public; these latter articles will be written by postdoctoral researchers under the supervision of our Editor Cornelia de Moor.

As a not‐for‐profit journal owned by the charity FEBS, *FEBS Open Bio* plays an important role in giving back to the scientific community. As part of this mission, I consider that the journal should support the annual FEBS Congress. The abstracts book has been published as a supplement in *FEBS Open Bio* for the last two congresses, and we look forward to continuing to serve the Congress in this capacity. In addition, we will present two different awards at the next Congress, to be held this year in Ljubljana. First, the *FEBS Open Bio* Article Prize will be awarded to an early‐career researcher (either a PhD student or a postdoctoral researcher within 5 years of receiving their PhD) who is the first author of a paper of especial interest and importance published in the journal in the preceding year, as decided by the Editorial Board. The prize consists of a bursary for registration, travel and accommodation to allow the winner to attend the FEBS Congress. Second, we will present prizes to the presenters of two outstanding speed talks at the FEBS Congress. A panel of jurors from the journal's Editorial Board will attend each shortlisted speed talk and judge it based on the interest and novelty of the work and the quality of the presentation. Each winner will receive €200, to be presented at the Congress Closing Ceremony.

Finally, we hope to mark the 10th anniversary of *FEBS Open Bio* in style with a special issue towards the end of the year. This issue will focus on the history and future direction of the journal and will feature articles from editors and authors of highly cited articles published in *FEBS Open Bio* over the last ten years.

## In closing

This has truly been an eventful year for *FEBS Open Bio*, with many successes and challenges. We have accomplished a lot this year, and I hope we can build on this success in 2021. None of this would be possible without the hard work of everyone on the journal. I extend my gratitude to our Editorial Board, all of whom have generously provided their time and expertise to handle so many submissions. I appreciate this was no small task for our editors, all of whom will have had many other urgent matters to attend to at this chaotic time. I would also like to thank our Editorial Manager Duncan Wright and Editorial Assistant Jacob Weller for their hard work and dedication to keeping the journal running smoothly at this tumultuous time of growth and disruption. I am indebted to Mary Purton, FEBS Press Publisher and formerly Executive Editor of *FEBS Open Bio*, who has continued to provide expert advice and guidance. I am also thankful for the great efforts of the nascent Editorial Advisory Board and all of our volunteer reviewers, whose hard work has enabled us to review so many submissions in 2020. I am very grateful to our Image Integrity Analyst Jana Christopher, whose immense powers of perception were really put to the test in this busy year. I extend my gratitude to Jackie Jones and all of our contacts at Wiley, who have provided excellent and considerate support all year. I would also like to offer my special thanks to Felix Wieland, the Managing Editor of *FEBS Letters* and Senior Editor of *FEBS Open Bio* since its launch. Felix will step down as Managing Editor this year, and I would like to congratulate him on his long‐standing and excellent service. I am also very grateful for the support and guidance of László Fésüs, who finished his nine‐year term as Chair of the Publications Committee at the end of 2020. László has worked tirelessly for FEBS Press, demonstrating strong commitment and leadership in the face of many challenges to the industry. I would like to warmly welcome Johannes Buchner as the incoming Chair of the Publication Committee, and I look forward to working with him to further improve the journal.

Last but certainly not least, I would like to thank all of our authors, reviewers and readers, without whom none of this would have been possible. I hope that *FEBS Open Bio* will continue to be among your journals of choice for publication and that we can work together as a community to ensure that sound science gets reviewed, published and promoted. I wish all of you and your friends, families and colleagues the very best for 2021 and would like to encourage you to submit your latest work to *FEBS Open Bio* in our 11th year of publication.

## References

[1] Alvarez RG , Karki P , Langleite IE , Bakksjø R‐J , Eichacker LA and Furnes C (2020) Characterisation of a novel cold‐adapted calcium‐activated transglutaminase: implications for medicine and food processing. FEBS Open Bio 10, 495–506. 10.1002/2211-5463.12826 PMC7137806 32115900

[2] Lerner A , Ramesh A and Matthias T (2020) The temperature and pH repertoire of the transglutaminase family is expanding. FEBS Open Bio 10, 492–494. 10.1002/2211-5463.12839 PMC7137796 32170837

[3] Horigane S‐I , Ozawa Y , Zhang J , Todoroki H , Miao P , Haijima A , Yanagawa Y , Ueda S , Nakamura S , Kakeyama M *et al* (2020) A mouse model of Timothy syndrome exhibits altered social competitive dominance and inhibitory neuron development. FEBS Open Bio 10, 1436–1446. 10.1002/2211-5463.12924 PMC7396430 32598571

[4] Gregory KS , Liu SM and Ravi Acharya K (2020) Crystal structure of botulinum neurotoxin subtype A3 cell binding domain in complex with GD1a co‐receptor ganglioside. FEBS Open Bio 10, 298–305. 10.1002/2211-5463.12790 PMC7050238 31945264

[5] Nam H , Hwang BJ , Choi D‐Y , Shin S and Choi M (2020) Tobacco etch virus (TEV) protease with multiple mutations to improve solubility and reduce self‐cleavage exhibits enhanced enzymatic activity. FEBS Open Bio 10, 619–626. 10.1002/2211-5463.12828 PMC7137792 32129006

[6] Majai GE , Gogolák P , Tóth M , Hodrea J , Horváth D , Fésüs L , Rajnavölgyi É and Bácsi A (2020) Autologous apoptotic neutrophils inhibit inflammatory cytokine secretion by human dendritic cells, but enhance Th1 responses. FEBS Open Bio 10, 1492–1502. 10.1002/2211-5463.12904 PMC7396436 32473089

[7] Solís C and Robinson JM (2020) Cardiac troponin and tropomyosin bind to F‐actin cooperatively, as revealed by fluorescence microscopy. FEBS Open Bio 10, 1362–1372. 10.1002/2211-5463.12876 PMC7327902 32385956

[8] Tsuji M (2020) Potential anti‐SARS‐CoV‐2 drug candidates identified through virtual screening of the ChEMBL database for compounds that target the main coronavirus protease. FEBS Open Bio 10, 995–1004. 10.1002/2211-5463.12875 PMC7262888 32374074

[9] Facchiano A , Facchiano F and Facchiano A (2020) An investigation into the molecular basis of cancer comorbidities in coronavirus infection. FEBS Open Bio 10, 2363–2374. 10.1002/2211-5463.12984 PMC7537529 32970391

[10] Voelkel S , Varga‐Atkins T and Mello LV (2020) Students tell us what good written feedback looks like. FEBS Open Bio 10, 692–706. 10.1002/2211-5463.12841 PMC7193163 32176832

[11] Lacey MM , Campbell SG , Shaw H and Smith DP (2020) Self‐selecting peer groups formed within the laboratory environment have a lasting effect on individual student attainment and working practices. FEBS Open Bio 10, 1194–1209. 10.1002/2211-5463.12902 PMC7327925 32438509

[12] Wanieck K , Ritzinger D , Zollfrank C and Jacobs S . (2020) Biomimetics: teaching the tools of the trade. FEBS Open Bio 10, 2250–2267. 10.1002/2211-5463.12963 PMC7609788 32860736

[13] Wang Y , Zhao Y , Wang H , Zhang C , Wang M , Yang Y , Xu X and Hu Z (2020) Histone demethylase KDM3B protects against ferroptosis by upregulating SLC7A11. FEBS Open Bio 10, 637–643. 10.1002/2211-5463.12823 PMC7137800 32107878

[14] Katiyar A , Fujimoto M , Tan K , Kurashima A , Srivastava P , Okada M , Takii R and Nakai A (2020). HSF1 is required for induction of mitochondrial chaperones during the mitochondrial unfolded protein response. FEBS Open Bio 10, 1135–1148. 10.1002/2211-5463.12863 PMC7262932 32302062

[15] Shirai T , Obara T and Takemasa T (2021) Effect of endurance exercise duration on muscle hypertrophy induced by functional overload. FEBS Open Bio 11, 85–94. 10.1002/2211-5463.13028 PMC7780094 33155405

[16] Ruf VC , Shi S , Schmidt F , Weckbecker D , Nübling GS , Ködel U , Mollenhauer B and Giese A (2020) Potential sources of interference with the highly sensitive detection and quantification of alpha‐synuclein seeds by qRT‐QuIC. FEBS Open Bio 10, 883–893. 10.1002/2211-5463.12844 PMC7193167 32190992

[17] Kai S , Matsuo Y , Nakagawa S , Kryukov K , Matsukawa S , Tanaka H , Iwai T , Imanishi T and Hirota K (2019) Rapid bacterial identification by direct PCR amplification of 16S rRNA genes using the MinION™ nanopore sequencer. FEBS Open Bio 9, 548–557. 10.1002/2211-5463.12590 PMC6396348 30868063

[18] Christopher J (2018) Systematic fabrication of scientific images revealed. FEBS Lett 592, 3027–3029. 30047985 10.1002/1873-3468.13201

[19] Byrne JA and Christopher J (2020) Digital magic, or the dark arts of the 21st century—how can journals and peer reviewers detect manuscripts and publications from paper mills? FEBS Lett 594, 583–589. 32067229 10.1002/1873-3468.13747

